# The complete chloroplast genome sequence of the medicinal shrub *Daphne Giraldii* Nitsche. (Thymelaeaceae)

**DOI:** 10.1080/23802359.2019.1644233

**Published:** 2019-07-22

**Authors:** Fang Yan, Xu Tao, Qin-Li Wang, Zhang Ya Juan, Chun-Mei Zhang, Hou Liang Yu

**Affiliations:** a Key Laboratory of Hexi Corridor Resources Utilization of Gansu, Hexi University, Zhangye, China;; b Engineering and Technical Research Center for Greenhouse Vegetable Production in Hexi Corridor, Hexi University, Zhangye, China

**Keywords:** *Daphne giraldii*, Thymelaeaceae, chloroplast genome, Illumina sequencing, phylogenetic analysis

## Abstract

*Daphne giraldii* Nitsche. (Thymelaeaceae) is a slow-growing shrub which has been used in Chinese folk medicine and commonly called ‘Zu Shima’. In this study, we assembled the complete chloroplast (cp) genome of *D. giraldii* using data from high-throughput Illumina sequencing. The *D. giraldii* cp genome is 171,643 bp in size and includes two inverted repeat regions of 41,798 bp each, which is separated by a large single copy region of 85,171 bp and a small single copy region of 2876 bp. A total of 137 genes were predicted, including 38 tRNA, 8 rRNA, and 90 protein-coding genes. In addition, 10 PCG genes possess a single intron, 92 PCG genes no intron, 1 gene harbor two introns. Six tRNA genes harbor a single intron. Phylogenetic analysis indicated that *D. giraldii* is closer to *Daphne kiusiana* and *Daphne tangutica* than other taxa.

*Daphne giraldii* Nitsche. (Thymelaeaceae) is a slow-growing medicinal shrub and it has been used in Chinese folk medicine, commonly called ‘Zu Shima’ (Huyiligeqi Dong et al. 2016). It often grows in virgin land on hillsides at elevations of 2000 m to 2500 m in the northwest areas of China (Su and Wu 2014; Yan et al. 2016). The wild resource of *D. giraldii* has been using up due to over-exploitation and deterioration of ecological environment (Geng et al. 2016). Therefore, a good knowledge of its population genetics would be useful for protecting wild resources of this plant. We aimed to assemble and characterize *D. giraldii’*s cp genome for researching the phylogenetic relationships of this plant and it would be essential to the conservation of this Endangered species.

Total genomic DNA was extracted from fresh leaves collected from in Gansu Longnan mountain regions (33°59′14″N, 104°48′6″E, 2240 m). A voucher specimen (K360-01-R206) is deposited at the Library No. 6, Sample Center, Shunjie Building, No. 12, Shunyi District, Beijing. A genomic library with an insert size of _400 bp was prepared using a TruSeq DNA Sample Prep Kit (Illumina, Beijing, China) and sequenced on an Illumina HiSeq X Ten platform. Approximately, 8 Gb of raw data were generated through pair-end 150 bp sequencing. After removal of adapter sequences, raw reads were fed into the NOVOPlasty (Dierckxsens et al. 2017) for assembly with the rbcL gene of *Daphne kiusiana* (GenBank accession *KY991380*) as the seed sequence. The assembled cp genome was annotated using the online annotation tool DOGMA (Wyman et al. 2004) and further corrected manually. The complete cp genome of *D. giraldii* (GenBank accession *MN080709*) was 171,643 bp in length, consisting of a pair of inverted repeat regions of 41,798 bp each, a large single copy region of 85,171 bp, and a small single copy region of 2876 bp. A total of 137 genes were annotated, including 38 tRNA, 8 rRNA, and 90 protein-coding genes. The overall GC content of the cp genome was 36.8%. In addition, 10 PCG genes (*rps16*, *rps12*, *atpF*, *rpoC1*, *petB*, *petD*, *rpl16*, *ndhA*, *ndhB,* and *rpl2*) possess a single intron, 92 PCG genes no intron, 1 gene (*ycf3*) harbor two introns. 6 tRNA genes (trnA-UGC, trnI-GAU, trnK-UUU, trnG-UUC, trnL-UAA and trnV-UAC) harbor a single intron.

We used RAxML (Stamatakis 2014) with 1000 bootstraps under the GTRGAMMAI substitution model to reconstruct a maximum likelihood (ML) phylogeny of 19 complete plastomes of Malvaceae, 1 one complete plastome of Bixaceae, 7 complete plastomes of Thymelaeaceae, 6 complete plastomes of Dipterocarpaceae, using *Brassica juncea* and *Morettia canescens* as outgroup. The phylogenetic analysis indicated that *D. giraldii* is closer to *Daphne kiusiana* and *Daphne tangutica* than other taxa in this study (Figure 1). Most nodes in the plastome ML trees were strongly supported. The complete plastome sequence of *D. giraldii* will provide a useful resource for the conservation genetics of this species as well as for the phylogenetic studies of Thymelaeaceaes.

**Figure 1.**
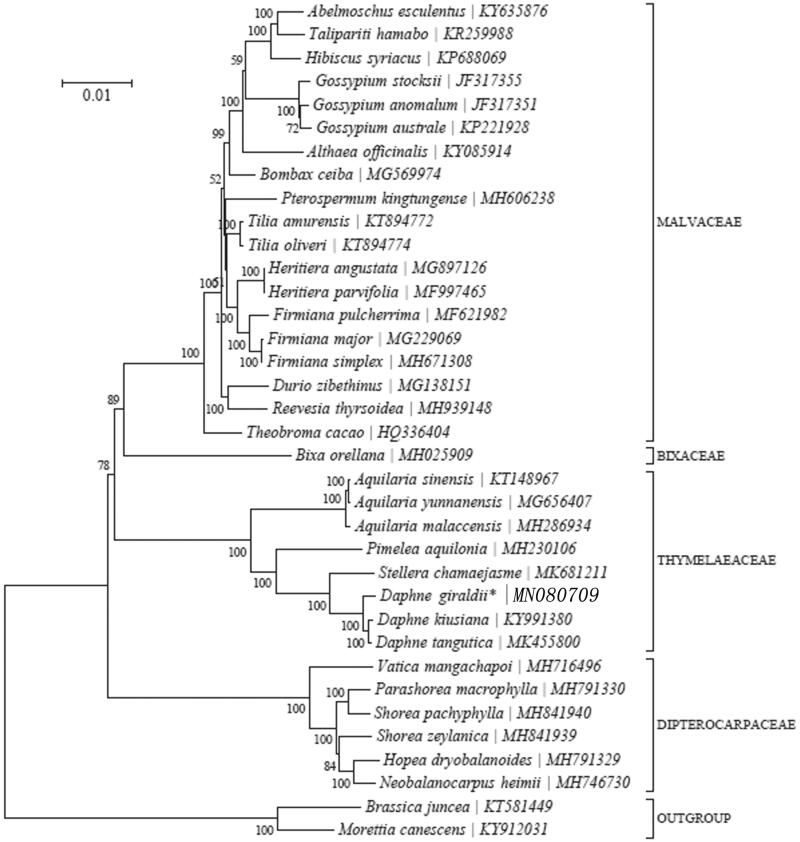
Maximum-likelihood (ML) tree of *D. giraldii* and its related relatives based on the complete chloroplast genome sequences.
